# Sexually-transmitted monkeypox: report of two cases^⋆^

**DOI:** 10.1016/j.abd.2022.08.002

**Published:** 2022-09-20

**Authors:** Paula Sian Lopes, Gabriela Roncada Haddad, Hélio Amante Miot

**Affiliations:** a Clínica Paula Sian Lopes, São Paulo, SP, Brazil; b Department of Dermatology, Faculty of Medicine, Universidade Estadual Paulista, Botucatu, SP, Brazil

**Keywords:** Monkeypox, Sexually transmitted diseases, Sexually transmitted diseases, viral

## Abstract

Monkeypox is an emerging infection that has spread to all continents since May 2022. It is caused by the zoonotic monkeypox virus, consisting of double-stranded DNA, belonging to the *Orthopoxvirus* genus of the Poxviridae family, which has high transmissibility, especially by contact with the skin, favoring its sexual transmission. This case report describes a same-sex male couple, both aged 28 years old, without comorbidities. In the index case, perioral and penile lesions started ten days before the consultation, with rapid progression and a high fever that started eight days after the appearance of the lesions. In the second case, the perioral lesions started three days after the partner; however, he remained afebrile. Both were isolated, treated with symptomatic measures, and, after ulceration, the lesions completely regressed in 14 days. Dermatologists should be aware of manifestations of monkeypox, which may include vesiculopustular lesions in areas of sexual contact, as well as oligosymptomatic cases or cases with few skin lesions.

In July/2022, the World Health Organization declared monkeypox (MP) a public health emergency of international importance, as cases have been reported in more than 80 countries, on all continents. At the beginning of August/2022, more than 2,100 cases, among adults and children, and one death in an immunosuppressed patient, were confirmed in Brazil.^1^ The monkeypox virus is an enveloped double-stranded DNA zoonotic virus that belongs to the genus *Orthopoxvirus* and the family Poxviridae. Sporadic outbreaks of MP have been reported in Africa since the 1970s, usually originating from contact with wildlife reservoirs, being currently endemic in sub-Saharan communities. With the eradication of smallpox (1980) and the discontinuation of vaccination, the world’s population became susceptible to zoonotic poxvirus infections, and MP emerged as a public health problem due to its high rate of contagion.^2^, ^3^, ^4^

MP has three stages in humans: incubation (seven to 14 days); general symptoms and rash.^5^, ^6^ The initial symptoms include fever, headache, asthenia, myalgia, and lymphadenopathy. After one to five days, a maculopapular rash appears, with centrifugal distribution. Lesions are 0.5‒1 cm in diameter and can range from a few and local to thousands, synchronously developing into four phases (macular, papular, and vesiculopustular with central umbilication, before ulcerating and resolving in 14–21 days).^2^

MP transmission can occur through large respiratory droplets, contact, and possibly contaminated fomites. However, it mainly occurs through contact with the lesions on the skin, and the most recent cases have occurred in men who have sex with men. Perineal and genital lesions suggest transmission by sexual intercourse and may be confused with herpes simplex, molluscum contagiosum, or syphilis.^2^, ^5^ Patient isolation should be maintained for 14–21 days and isolation of close contacts for 14 days.^6^ The risk factors for MP include being a young male, and risk behaviors such as sexual activity without a condom, with many partners, and a history of sexually transmitted infections.^4^, ^6^, ^7^ This case report describes a same-sex male couple, who showed all these risk factors and developed sexually transmitted MP.

## Index case

**Figure 1:**
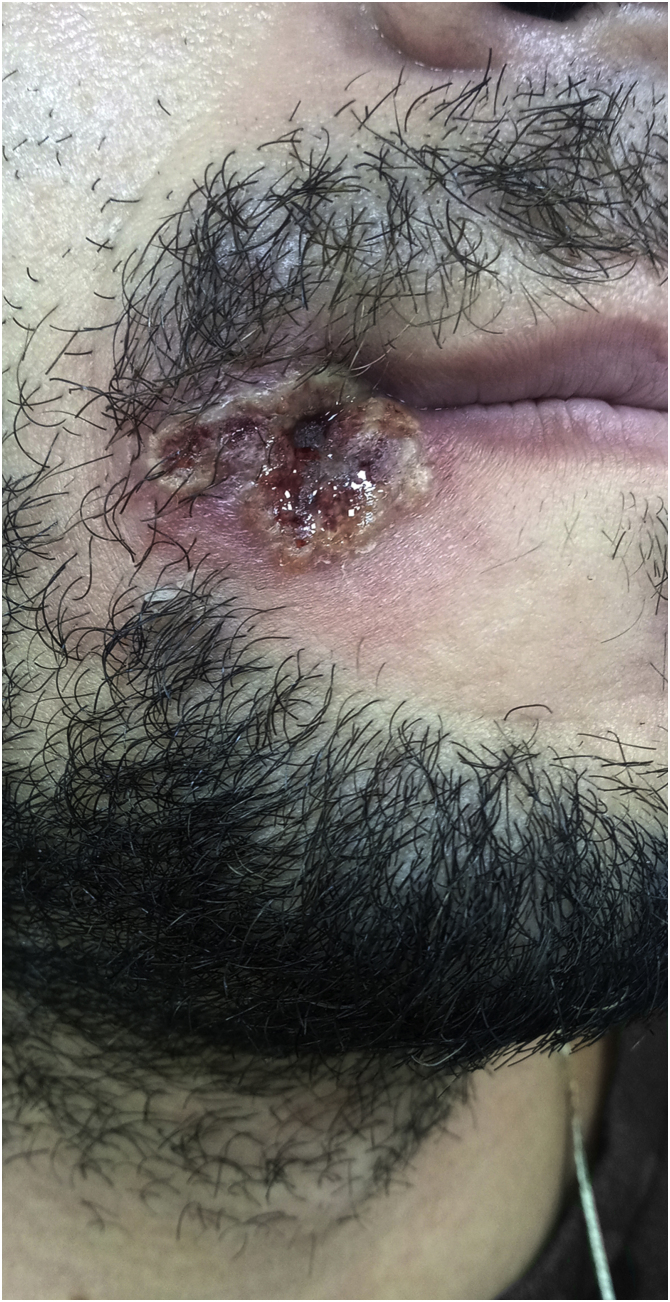
Monkeypox. Confluent pustules outlining central umbilication, and central ulcero-necrotic area, located in the right labial angle.

**Figure 2:**
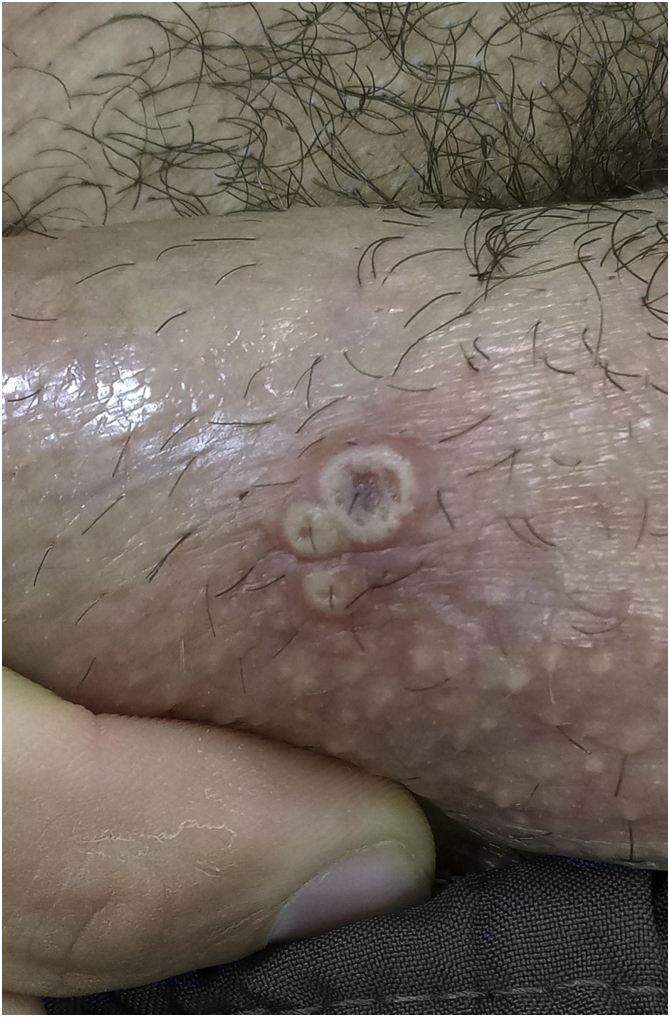
Monkeypox. Three pustules with central umbilication on the shaft of the penis.

A 28-year-old male patient reported “a sore” in the corner of the mouth for ten days, with rapid progression, accompanied by cervical lymphadenopathy, myalgia, headache, and fever (39 °C) eight days after the initial appearance of the lesion. He reported having sex with other partners without a condom, or the regular use of pre-exposure prophylaxis to prevent HIV infection. He also mentioned effective treatment for latent syphilis two months before. On examination, he was apathetic, had an ulcero-crusted lesion, with active vesiculopustular borders, measuring 1.5 cm on the rima oris, with a necrotic, exudative background, and local erythema (Fig. 1), besides painful and hardened cervical and supraclavicular lymphadenopathy, measuring 2 cm in diameter. He also had three umbilicated pustules measuring 3 mm on the shaft of the penis, and inguinal lymphadenopathy (Fig. 2).

## Case 2

**Figure 3:**
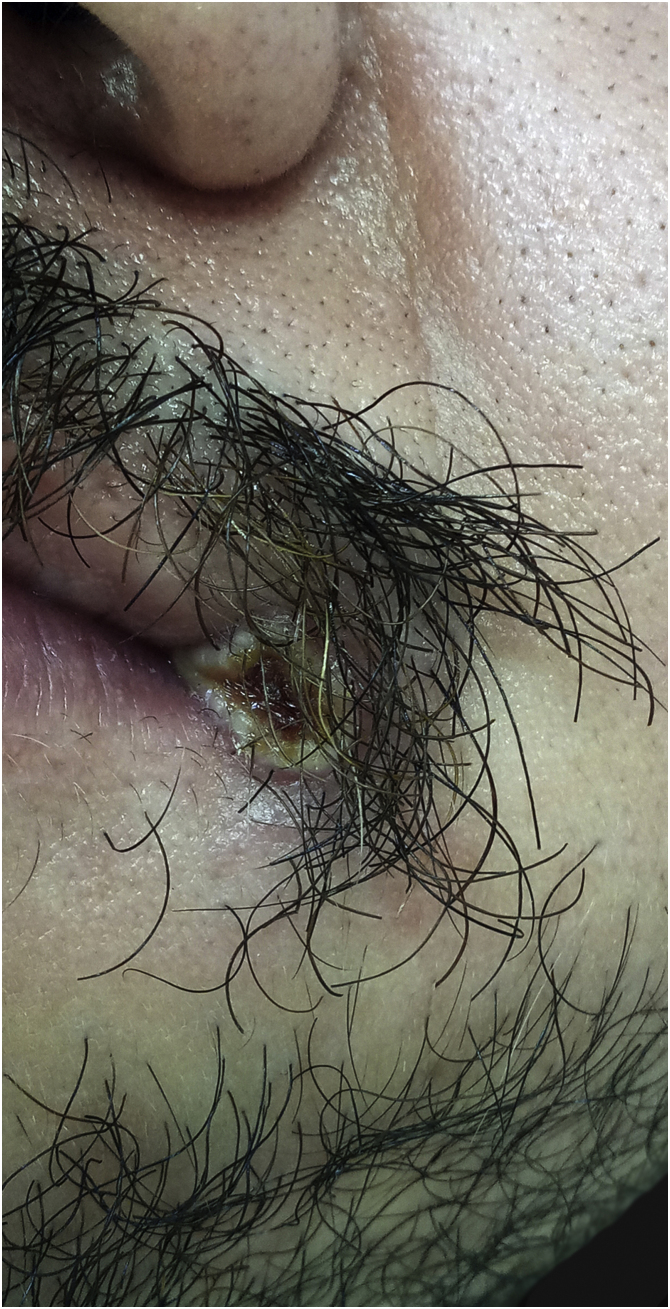
Monkeypox. Pustule with a depressed and necrotic center located at the left labial angle.

The 28-year-old male partner was in a stable relationship with the index case; he was asymptomatic, and reported a “lesion” on the angle of the mouth for a week. He has also been treated for latent syphilis two months before and reported regular use of pre-exposure prophylaxis to prevent contamination with HIV. On physical examination, he was in good general condition, with submental lymphadenopathy (2 cm, indurated, painful and mobile), without genital lesions. He had a 6-mm lesion on the left rima oris with active vesiculopustular borders, central necrotic tissue and an erythematous base (Fig. 3).

The hypotheses of herpes simplex and MP were raised. The blood count showed lymphocytosis, HIV serology was negative, and the non-treponemal test (VDRL) was 1/8. The real-time PCR (lesion swab), performed at Fleury Laboratory (São Paulo), confirmed the diagnosis of MP in both individuals.

The patients refused to undergo a biopsy of the lesions due to trypanophobia and were treated with paracetamol, instructed to isolate from contacts until the lesions healed, and clean the lesions with antiseptics. After 14 days the lesions regressed leaving residual dyschromia. In a series of 528 MP cases from 16 countries, 98% of those infected were men who had sex with men, with a median age of 38 years. Cutaneous manifestations were identified in 95% of cases. The anogenital area (73%), trunk and limbs (55%), and face (25%) were the most affected areas. Lesions on the palms and soles were reported in only 10% of cases; however, mucous membranes were affected in 41%, and isolated proctitis (14%) was also reported.^2^ Fever (62%), painful lymphadenopathy (56%), headache (27%), prostration (41%), and myalgia (31%) were the most frequently reported symptoms. Cutaneous manifestations are evident in MP, and the role of the dermatologist in the early diagnosis of skin lesions is essential in controlling the outbreak. However, in 47% of the cases, the lesions affect up to two regions of the body, which causes some diagnostic confusion, especially with other sexually transmitted diseases, mainly in a group of individuals with risk behavior for sexual transmission.^8^, ^9^ MP is a self-limiting disease and the overall mortality has been low in this recent global outbreak (<0.1%). However, very young children and those with immunodeficiencies may have more severe clinical manifestations, such as encephalitis, keratitis, myocarditis, epiglottitis, and pneumonitis; or secondary bacterial infections.^2^, ^9^, ^10^ The screening of the lesions by PCR is becoming widely available in laboratories and hospitals in major urban centers, and the histopathological examination of the lesions may suggest the diagnosis, in addition to help exclude other differentials.

Dermatologists should be aware of vesiculopustular lesions in areas of sexual contact and mucous membranes (anogenital and oral regions) as possible manifestations of MP, as well as oligosymptomatic cases or with few cutaneous lesions.

## Financial support

None declared.

## Authors' contributions

Paula Sian Lopes: Planning of the study, writing and approval of the final version of the manuscript.

Gabriela Roncada Haddad: Planning of the study, writing and approval of the final version of the manuscript.

Hélio Amante Miot: Planning of the study, writing and approval of the final version of the manuscript.

## Conflicts of interest

None declared.
